# Preformed pediatric zirconia crown versus preformed pediatric metal crown: study protocol for a randomized clinical trial

**DOI:** 10.1186/s13063-019-3559-1

**Published:** 2019-08-24

**Authors:** Serena Lopez-Cazaux, Elody Aiem, Ana Miriam Velly, Michèle Muller-Bolla

**Affiliations:** 1 Faculty of Dentistry, Department of Pediatric Dentistry, Unité d’Investigation Clinique en Odontologie (Uic11), University and Hospital of Nantes, 1 place Alexis Ricordeau, BP 84215, 44042 Nantes Cedex 1, France; 2 Department of Pediatric Dentistry, Faculty of Dentistry, UNS-UCA, CHUN, Nice, France; 3 Dental Department of Jewish General Hospital, Faculty of Dentistry, McGill University, Montréal, Canada; 4 Department of Pediatric Dentistry, Faculty of Dentistry, Université Côte d’Azur, Centre Hospitalier Universitaire de Nice, Nice, France; 5 Laboratory URB2i – EA 4462, University Paris Descartes, Paris, France

**Keywords:** Primary molar, Zirconia pediatric crown, Stainless steel crown, Carious lesion, Structural anomaly

## Abstract

**Background:**

Guidelines in pediatric restorative dentistry recommend the use of preformed pediatric stainless steel crowns (SSCs) in cases of severe tooth decay of at least two surfaces. This clinically effective and safe restorative option is frequently refused by parents for esthetic reasons; they prefer conventional restorations using esthetic filling materials (composites, glass ionomer) if lesion severity limited to two surfaces permits. Recently, manufacturers have proposed esthetic preformed pediatric zirconia crowns (ZCs) but these have been assessed in only two randomized clinical trials (RCT) with follow-ups of 6 and 12 months. Only one of these RCTs was carried out on primary molars to test ZCs (NuSmile ZR) without a groove in its inner surface. The primary objective of this proposed RCT is to assess the effectiveness of ZCs compared with SSCs. Our hypothesis is that the effectiveness of ZCs will be equivalent to that of SSCs.

**Methods:**

In this split-mouth, 2-year RCT, pairs of primary molars in 101 child participants will be randomized and restored with SSCs (ESPE, 3M) and ZCs (EZCrowns, Sprig Oral Health Technologies) characterized by grooves on their inner surface. Primary molars will first be allocated to SSCs, and 1 to 2 weeks later the other primary molar of the same pair will be restored by ZC. The primary outcome is the success defined by the “absence of major clinical and radiographic failure” (e.g., pain, pulp infection, dental abscess or periradicular pathology visible on radiographs). The secondary outcomes include the retention and fracture rates, the gingival condition, the wear of the antagonist of the treated teeth, as well as both parental and child satisfaction.

**Discussion:**

This study will investigate two types of preformed pediatric crowns for the management of severe decay on primary molars. The results may help practitioners choose the better therapeutic option and to explain to parents the advantages and disadvantages of these two therapies.

**Trial registration:**

NCT03296709. Registered on  27 September 2017.

**Electronic supplementary material:**

The online version of this article (10.1186/s13063-019-3559-1) contains supplementary material, which is available to authorized users.

## Background

For many years, the preformed pediatric metal crown (stainless steel crown (SSC)) was considered, without established scientific evidence, the best method of restoring primary molars (PMs) affected by severe carious lesions because of its higher longevity than conventional restorations using various filling materials (amalgams, composites, glass ionomer) [1]. In 2008, SSCs were recommended by the British Society of Pediatric Dentistry [2]. In 2015, a systematic review assessed the effectiveness and the safety of the various types of preformed pediatric crowns (PPCs) compared with various types of filling material (amalgam, composite, glass ionomer). This systematic review evidenced the effectiveness of SSCs and the authors considered them the most appropriate restorative technique when compared with traditional methods (relative risk = 0.18; 95% confidence interval (CI) 0.06–0.56) over a 12- to 24-month period [3]. In this systematic review, effectiveness was defined as the absence of “major failure,” defined as a composite measure of signs and symptoms leading to diagnosis of irreversible pulpitis or periradicular periodontitis (pain, pulp infection, dental abscess, periradicular pathology visible on radiographs). Henceforth, SSCs were proposed as the sole technique for coronal restorations by one of the few associations who propose good practice recommendations taking into account the evidence base—the American Association of Pediatric Dentistry—when carious lesions or structural anomalies affect more than two surfaces of the PMs. SSCs are one of the techniques recommended for coronal restorations if two surfaces are involved [4]. Unfortunately, SSCs are frequently refused by parents for esthetic reasons [5]. Recently, some manufacturers—Hass Corporation (Gangwon-do, Korea), Kinder Krowns (St Louis Park, MN, USA), NuSmile (Houston, TX, USA), and Sprig Oral Health Technologies (Loomis, CA, USA)—have proposed esthetic PPCs called zirconia crowns (ZCs).

Another systematic review [6] published in 2016 having the primary objective of comparing the effectiveness of PPCs by including at least one group of esthetic crowns did not identify any randomized clinical trial (RCT) that compared ZCs with one of the other categories of PPCs on the PMs.

Since 2016, only two RCTs were identified on https://clinicaltrials.gov and http://apps.who.int/trialsearch/Default.aspx; one published [7] and the other not yet completed. The published RCT (NCT03067337, first submitted 22 January 2017) assessed the effectiveness of ZCs (NuSmile) compared with SCCs. This RCT reported that the effectiveness of ZCs was similar to that of SCCs in terms of cracks, chips, and fractures. However, gingival and plaque indices were lower among ZCs than SCCs. Even if this RCT suggests that ZCs are better than SCCs, caution is necessary in interpreting the results. First, the power of the analysis comparing cracks, chips, and fractures between ZCs with SCCs may be lower since the author justified the sample size based only on the gingival index. Second, it was also unclear how many teeth per participant were included since the authors reported that a sample of 120 contralateral molars in 26 patients had been treated [7]. The incomplete RCT (NCT01919515) comparing ZCs (NuSmile ZR) with SSCs in PMs is noted to be without recruitment since 2014.

**Fig. 1:**
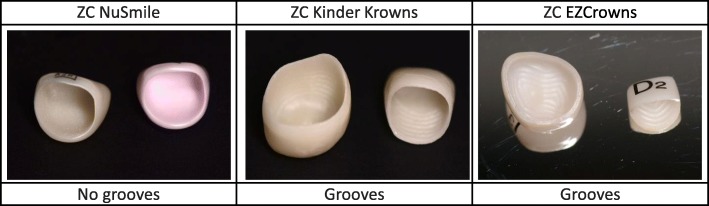
Characteristics of different zirconia crowns (ZCs)

ZCs manufactured by NuSmile differ from other ZCs by having no grooves on their inner surface. In order to improve retention and to absorb stresses in ZCs, other manufacturers (Kinder Krowns, Sprig Oral Health Technologies) have integrated grooves in the inner occlusal and axial surfaces of the PPCs (Fig. 1). Theses grooves are wider in EZCrowns (Sprig Oral Health Technologies). No RCT has so far evaluated these particular ZCs characterized by large inner surface grooves (EZCrowns). Thus, a split-mouth RCT comparing the effectiveness of ZCs (EZCrowns) and SSCs, considered as the reference PPC for PMs [2–4], must be carried out.

## Objectives and hypotheses

The overall aim of this RCT is to investigate the effectiveness of a new esthetic therapeutic option (EZCrowns; Sprig Oral Health Technologies) for the management of dental caries or structural anomalies. More specifically, the primary objective of this RCT is to assess the success of ZCs in PMs in comparison with SSCs with a follow-up of 2 years. The control treatment will be SSCs since this is the standard crown option [2–4].

Due to the esthetic superiority of ZCs (being a natural dental color) in comparison with SSCs (being metallic), our working hypothesis is that the success rates are equivalent between the ZCs and SSCs, given the absence of any major failure defined by Innes et al. [3] as a composite criterion including clinical and radiographic criteria.

The secondary objectives are to evaluate the retention and fracture rates, the gingival condition (plaque index, gingival index and depth of the pocket of Löe and Silness [8]), the wear of antagonist teeth [9, 10], and parental and child satisfaction (using a Likert scale) [11] between the study groups during the same period.

## Methods

### Trial design and blindness

**Fig. 2:**
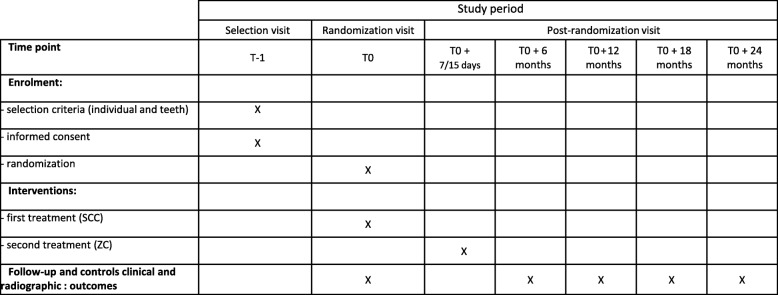
Participant's timetable . SCC stainless steel crown, ZC zirconia crown

In this multicenter split-mouth RCT, a PM and its contralateral tooth in the same arch will be randomly assigned to a specific crown treatment group, ZC or SSC (Fig. 2). We plan to screen 101 individuals to successfully recruit 81 patients (see Sample size section below). We decided to use the split-mouth design because each subject forms its own control, adjusting for potential confounders.

We will blind the statistician supervising the analyses because it is feasible and it reduces the risk of certain biases (e.g., detection bias). It is not possible, however, to blind patients, operators, or outcome examiners because each crown treatment (ZC and SSC) has its own specific color.

### Randomization

Each tooth of the pair (two contralateral PM in the same arch) in the patient will receive one of the two treatments. PMs will first be allocated to SSCs, which correspond to the gold standard treatment; 1 to 2 weeks later, the contralateral PM of the same pair will be restored by ZCs (e.g., if an SSC is assigned on the right PM, the left tooth of the pair will be treated with a ZC). This randomization method focusing first on SSCs has the advantage of being without consequence for one of the secondary outcomes (parental and child satisfaction). The randomization will be performed using a computerized and centralized system at the Nice University Hospital (CHUN) via a specific website (INCLUSIO, https://inclusio.chu-nice.fr) after inclusion of the subject. The sequence of randomization will be stratified by center and dental arch.

### Study population

The participants and dental eligibility criteria are described in Table 1. Dental exclusion criteria have been chosen to reduce failure that is independent of the treatment studied. No special concomitant dental care or intervention is prohibited in children after inclusion in the trial, except that concerning the included teeth.

**Table 1:** Eligibility criteria

Inclusion criteria	
• Child aged 4–13 years old in good general health (American Society of Anesthesiologists (ASA) class I or II)	
• Child covered under health insurance of their parent(s)	
• Child and parent(s)/legal guardians speak French	
• Consent of parents/legal guardians and child	
• Child cooperative in the vigilant or sedated state (Venham score of 0, 1, or 2) [12]	
• Child with at least two primary molars (PMs) of the same type (first or second PMs) contralateral (fractional mouth) meeting the following criteria:	
– the two PMs of the pair (54–55, 64–65, 74–75 or 84–85) are affected by a cavitated carious lesion or a hypoplastic defect on at least two surfaces.	
– both PMs of the pair may or may not have a pulpotomy prior to the placement of the preformed pediatric crowns	
– each PM of the pair must have an antagonist tooth	
General exclusion criteria	
• Noncompliant child for dental care in the vigilant state or under conscious sedation, who must therefore be treated under general anesthesia	
• Child allergic to local anesthesia, chromium, or nickel	
Dental exclusion criteria	
• Severely decayed PM prohibiting the retention and the sealing of the restoration	
• PM with spontaneous pain	
• PM with exposed cement or with evidence of swelling in the surrounding tissues	
• PM in infraclusion	
• PM whose radiographic examination reveals a widening of the desmodontal space, the presence of radiolucent image on the root furcation and/or apices, internal or external resorption	
• PM whose physiologic exfoliation will appear within 24 months or PM with a root resorption of more than a third of the radicular length	

### Recruitment procedures

**Fig. 3:**
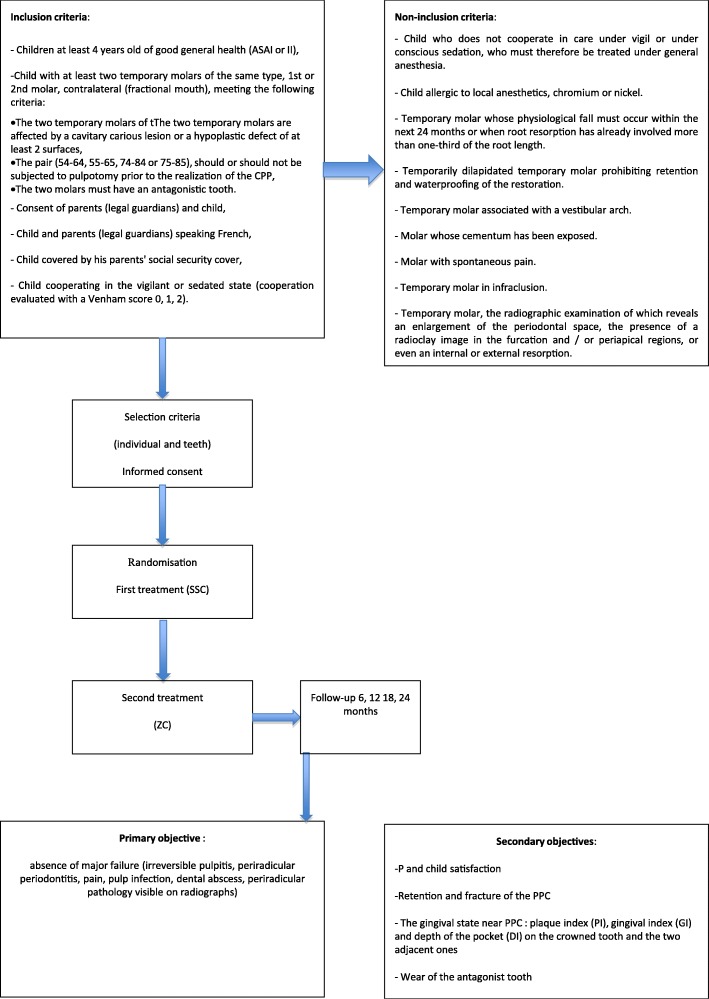
Study flow chart. ASA American Society of Anesthesiologists, P parent, PPC preformed pediatric crown, SCC stainless steel crown, ZC zirconia crown

Participants will be recruited in nine Departments of Pediatric Dentistry in the university hospitals of Bordeaux, Lille, Nancy, Nantes, Nice, Paris Bretonneaux, Paris Louis Morrier, Strasbourg, and Toulouse. Each center will recruit an average of 12 children (inclusion from September 2018 to February 2020). The participant's timetable is shown in Fig. 2 and the flow chart of the study is shown in Fig. 3. To encourage recruitment in order to reach the target sample size in the 18-month period, the principal investigator (MM-B) will send a text message of encouragement, with general center recruitments mentioned after each inclusion.

At the first visit, eligibility criteria (Table 1) will be verified by the operator in each center after the clinical and radiographic examination. Potential eligible participants and their legal guardians will receive information about the study. The inclusion will then be formalized after legal guardians and the child sign the informed consent forms.

### Intervention

At the second visit, the randomization will take place. The center operator will then perform the restoration with an SSC (ESPE; 3M, Paris France). The tooth is anesthetized, demineralized dentine will be removed and, if a pulpotomy is needed, the rubber dam must be placed on the PM to perform the treatment (pulpotomy with IRM; Dentsply Sirona, Charlotte, NC, USA) and the subsequent restoration is with high-viscosity glass ionomer cement (GIC) (Equia Forte; GC, Leuven, Belgium). The tooth will be prepared by occlusal and proximal reduction; a SCC will be tried and adjusted to fit the tooth. Occlusion is checked, and the SSC will be sealed with a GIC (CVI Fuji Plus; GC).

At the third visit (1 to 2 weeks later), the operator will undertake restoration of the other PM with a ZC (EZCrown; Sprig Oral Health Technologies). After anesthesia and excavation of demineralized dentine, a pulpotomy will be performed only if it was indicated on both PMs of the pair (pulpotomy after rubber dam placement with IRM; Dentsply Sirona). After the subsequent restoration with high-viscosity GIC (Equia Forte; GC), the PM will be prepared following the manufacturer’s guidelines by occlusal, peripheral, and infra-gingival reduction. The ZC will be tried and occlusion will be checked, and the ZC will be sealed with a GIC (CVI Fuji Plus; GC).

The operators (one per center) have followed a course organized by SL-C (using videos) and received training on Typodont models on how to prepare teeth for ZCs.

### Study outcomes

The primary outcome is the success of the treatment. Success will be defined by the absence of major failure. A composite measure of signs and symptoms leading to diagnosis of irreversible pulpitis or periradicular periodontitis will be used to define major failure. This composite measure includes one or more of the following: pain, pulp infection, dental abscess, and periradicular pathology visible on radiograph [3].

The secondary outcomes are parental and child satisfaction (size, form, and color), retention and fracture of the PPC (two dichotomized variables, yes/no), the gingival state near the PPC using the indices Löe and Silness to record plaque index (PI), gingival index (GI) and depth of the pocket (DI) on the crowned tooth and the two adjacent teeth [8].

Parental and child satisfaction will be evaluated on a five-point Likert scale [11]. The DI will be assessed by soft periodontal probing on the sulcus mesial, distal, vestibular and lingual areas of the teeth. Finally, the wear of the antagonist tooth will be recorded (0: absence of wear; 1: wear on only the cusp point; and 2: wear at least at the cusps) [9, 10]. The presence or absence of a restoration with a dental material of the antagonist tooth will be also recorded.

Primary and secondary outcomes will be assessed clinically and radiographically by independent evaluators, one per center, at different periods during the study (postintervention and every 6 months during a total follow-up of 2 years). To prevent information bias, the evaluators will not be the operators who applied the treatment.

### Assessment and data collection

#### Baseline visit

At the first visit, before treatment commences, participant characteristics (e.g., age and sex), the numbers of teeth included (54–55, 64–65, 74–75, 84–85), the number of surfaces affected by the carious lesion or the hypoplastic defect, and the pulpotomies indicated in both teeth, will be recorded by the nine operators (one per center).

#### Follow-up visits

After 6, 12, 18 and 24 months of follow-up, the primary (success criteria) and secondary outcomes (see Study outcomes section above) will be assessed by the nine evaluators (one per center and not the operator in each center).

To improve internal validity, evaluators (one per center) will be trained and calibrated on how to assess the study outcomes by means of repeated exercises on the Socrative website (https://socrative.com/) with clinical cases presented in a random way using the model of e-calib [13]. These exercises as prepared by Dr Clara Jopeph (Department of Pediatric Dentistry, Faculty of Dentistry, Nice, France), SL-C, EA, and MM-B will allow the evaluation of the inter- and intra-evaluator agreement (Kappa coefficients).

### Data management

The baseline data, follow-up trial data, and adverse events will be recorded by operators and evaluators on case report forms (CRFs). Data will be kept anonymous. Patients will be identified by their inclusion data; only the number of the patient, number of the center, and the initial letter of their first and last name will be registered on the CRF.

### Sample size

Table 2 shows the sample size calculation for success rates ranging from 95% to 97%, alpha equal to 5%, with 90% power and an equivalence margin between the study groups of 10%. This table illustrates that the effective sample size of 81 teeth per treatment group will provide a power of 90% to assess effectiveness equivalence between the treatment groups. These rates were based on the systematic review of Innes et al. [3]. This calculation was based on the formula (Z _0.95_ + Z _0.90_)^2^ [Ps (1 – Ps) + Pn (1 – Pn)] / (Ps – Pn – D)^2^ (see http://people.ucalgary.ca/~patten/blackwelder.html) [14].

**Table 2:** Sample size calculation

Rate	Teeth	Total required
80	274	548
85	218	438
90	154	308
95	81	162
97	49	98

Note: Rate of the outcome for the study groups, power = 90%, alpha = 5%, equivalence margin = 10%

Additionally, when we consider a hazard ratio (HR) = 1, moderate intraclass correlation (ICC) = 0.05, the percentage of teeth in each study group of 50%, the probability of observing an event of 90%, and two one-sided test (TOST) Zα = 1.64, this sample size of 81 teeth provides 80% power to test the equivalence between the study groups for margins ranging from 0.7 to 0.9. This sample size was calculated as described in [15].

Furthermore, assuming a dropout around 20%, the total sample size necessary will be 101 children. This dropout value was based on the experience of the research team from previous RCTs conducted in France [16]. This sample size is higher than those of past RCTs assessing the effectiveness of esthetic PPCs, which included 11 to 60 teeth per treatment group [6, 7].

### Statistical analysis

The demographic and clinical characteristics of the participants (center, gender, age, Venham score 0–2/3–5, vigilant state/conscious sedation) and tooth pair (maxillary/mandibular molar pair, carious lesion/hypoplastic defect, two or more surfaces affected, with or without pulpotomy) will be described using mean and standard deviation (SD) for quantitative variables and percentages for qualitative variables.

Furthermore, bivariate analysis will be used to compare the baseline characteristics between groups (e.g., left/right molars, presence or absence of a restoration with a dental material on the antagonist tooth).

An intent-to-treat (ITT) analysis will be conducted. To assess the equivalent effectiveness of ZCs, we will use a multivariable Cox hazards regression model with shared frailty including success as the dependent variable (primary outcome defined by the absence of major failure: no = 0, yes = 1), and the treatment group variable as an independent variable. This analysis will also include the potential confounders age, gender, type of tooth (i.e., first or second primary molar), pulpotomy, number of surfaces affected by the lesion, and presence or absence of a restoration with a dental material of the antagonist tooth. Cox proportional hazards models, using the PHREG procedure will estimate the HR and the 95% CI. In these analyses, a random statement will be included identifying the variable “subject” as the variable that represents the clusters.

Secondary analysis will be performed to compare: the secondary outcomes of retention and fracture of PPCs; PI, GI, and DI on the crowned tooth and the two adjacent teeth; presence or absence of a restoration with a dental material of the antagonist tooth; and wear of the antagonist tooth between treatment (ZC) and control (SSC) groups.

Both the ITT and secondary analyses will be performed within a 2-year period at 6, 12, 18, and 24 months. Repeated measures analysis (mixed model approaches) will be performed, and the principal independent variables will be treatment and time. The alpha level will be equal to 5%. All analyses will be performed with SAS (version 9.4; Cary, NC, USA).

The intra- and inter-investigator agreements for primary and secondary outcomes will be assessed using weighted Cohen’s Kappa. After calibration, the evaluators (one per center) will be tested by repeated exercises on the Socrative website (https://socrative.com/) with clinical cases suggested in a random way on the e-calib model (http://zep01793.dent.med.uni-muenchen.de/moodle/). Different clinical pictures were assessed twice, 15 days later.

### Data monitoring, harm, and auditing

The various centers will send their data to the coordinating center and data will be entered by the coordinating center. The data will be monitored by an independent clinical research assistant (MM-B; Clinical Research Direction, CHUN).

No interim analysis is planned. Serious and nonserious adverse events will be assessed during the study by the evaluators. Trial management may be audited by the French Department of Health at any time; the audit would be independent of the investigators and sponsor. Investigators will not have access to the final trial dataset, which will be assessed by the blinded epidemiologist AMV following the recommendations of the statistician.

### Ethical consideration

The Clermont-Ferrand Committee of Protection of the People (CPP; Sud-Est VI) has approved the study protocol in February 2018 (2017-A01952–51). The protocol is registered with the IDRCB (2017-A01952–51) at the French National Agency for Medicines and Health product Safety (ANSM) and in Clinicaltrials.gov (NCT03296709). All amendments to the protocol will be justified and submitted to the scientific board, accepted by the CPP, and recorded by the ANSM. Changes and amendments will be also recorded at ClinicalTrials.gov. Informed consent will be obtained from each eligible child and their legal guardians after an explanation of the trial by an investigator of the corresponding center. Patients and legal guardians are informed that they have the right to withdraw from the study at any time and without giving reasons. Regardless of withdrawal, patients will be provided with any necessary treatment in their best interest. Withdrawal will be documented. Data confidentiality was audited by the National Committee of Informatics and Freedom (CNIL; reference methodology 001). First and last names of included patients are not recorded in the database. Neither additional visits nor complementary examinations are needed compared with the conventional caries management for children at high risk of caries (our target population). However, the consultation may take a little longer because of the indicators used and the data collection. These methods of monitoring due to the research requirements involve only negligible constraints.

### Dissemination of the results

The Consolidated Standards of Reporting Trials (CONSORT) guidelines will be used in the preparation of the manuscripts reporting the results of this RCT, and the results will be published in international peer-reviewed journals [17]. Authors of the publications will be people involved in the elaboration of the protocol, the implementation and conduct of the trial, and the writing of the manuscript and report. A summary of the study results will be posted at ClinicalTrials.gov to allow general access to the findings. Data sharing will be at the participant level. Access to the full protocol can be granted to anyone upon request.

### Possible problems

One of the possible problems with our RCT will be the sample size because it may be difficult to recruit children with equivalent lesions in each pair of PMs. To account for this potential problem, the study sample will be recruited from nine teaching hospitals.

### Responsibilities

The coordination center as a member of the sponsor organization is responsible for overall data management, monitoring and communication among all sites, and general oversight of the conduct of the project. The investigator as a member of the sponsor organization is responsible for submitting the report for publication.

The coordination center is accredited by the European Clinical Research Infrastructure Network (https://www.ecrin.org).

## Discussion

At present, the success and reliability of SSCs is known [3]. Even if SCCs are recommended in the treatment of severe tooth decay in children, few dental practitioners adopt their use in clinical practice; one of the reasons for this is their poor esthetic appearance [5]. An esthetic alternative to SSCs is ZCs. They would be more widely adopted by both clinicians and policy makers if the evidence showed that their success rate is equivalent to the success rate of SCCs. ZCs are supported by only a few valid studies evaluating their effectiveness and reliability [7]. To date, only a few case series focusing on restoration of PMs with ZCs have been published; these concluded that they perform well [18–23]. There are no prospective clinical trials using a search of PubMed on the performance of ZCs on PM. Only one RCT with a high risk of bias has been carried out in 26 children with a follow-up of 12 months [7]. The establishment of a suitably powered clinical trial comparing SSCs and ZCs is essential, and an evaluation of the long-term success of ZCs compared with SCCs is required. This trial may help address this issue as it involves nine centers nationwide and the possible recruitment of a large sample of 101 patients. In addition, the inclusion criteria are broad and thus there is variation in the patients included, especially in terms of the individual risk of caries. Hence, the external validity of the data should be optimized.

All current recommendations from the Standard Protocol Item Recommendations for Interventional Trials (SPIRIT) statement were taken into account in the design of the present clinical trial figure [24] (see Additional file 1). In terms of internal validity, the sources of bias are limited by the use of centralized randomization (selection bias) and strict prospective data recording and monitoring (information bias). However, because of the color of the PPCs, the operators, patients, and evaluators cannot be blinded. We selected the split-mouth design for this RCT because it enabled us to control for potential confounders that could distort the results.

The choice of assessment criteria for the primary outcome is based on the systematic review of Innes et al. [3]. Using the same composite criteria will allow this RCT to be included in a future meta-analysis to compare different PPCs; their choice is more appropriate than the US Public Health Service criteria that is used more for the assessment of dental fillings. The success rate of a restoration can be related to the survey rate (presence in the mouth) of the tooth after 2 years of follow-up. This has the advantage of being longer than the 6 or 12 months of follow-up used in other studies focusing on PPCs [6, 7]. Other aspects of the criteria are also important and guide the choice of the criteria for the secondary outcome. The success of a restoration also includes its retention and integrity, the health of surrounding structures, and the satisfaction of both patients and parents with the restoration.

The clinical procedure is carried out under optimal conditions. One of the main advantages is that, if a pulpotomy must be done on the two PMs of the tooth pair, it has to be done under rubber dam isolation. Furthermore, the two PMs must present similar decay in depth, but this is often the case in children.

With sustained poor oral health in most developed communities [25], and greater importance being given to esthetic factors, the results of the present RCT will inform clinicians regarding the selection of clinical treatment options for severe dental decay which affect most children. The results will help them explain to the parents the advantages and disadvantages of the two possible solutions. The results will allow for advancement in the recommendations, and will be beneficial for the patient, the practitioner, and the public healthcare system.

## Trial status

This is version V1.2 of the protocol dated 20 October 2017.The recruitment began on 10 September 2018 and will end on 10 March 2020.

## Additional file

Additional file 1 : The SPIRIT checklist. (DOC 158 kb)

## Data Availability

Results are expected to be published in the medical literature and will be available for enrolled patients who wish to learn about the outcome.

## Abbreviations

ANSM: Agence Nationale pour la Sécurité du Médicament
CHUN: Centre Hospitalier Universitaire de Nice
CNIL: Comité National Informatique et Liberté
CONSORT: Consolidated Standards of Reporting Trials
CPP: Committee of Protection of the People
CRF: Case report form
DI: Depth of the pocket
GI: Gingival index
GIC: Glass ionomer cement
IFRO: Institut Français pour la Recherche en Odontologie
ITT: Intent-to-treat
PI: Plaque index
PM: Primary molar
PPC: Preformed pediatric crown
RCT: Randomized clinical trial
SPIRIT: Standard Protocol Item Recommendations for Interventional Trials
SSC: Stainless steel crown
ZC: Zirconia crown
